# Growth of a Large, Single-Crystalline WS_2_ Monolayer for High-Performance Photodetectors by Chemical Vapor Deposition

**DOI:** 10.3390/mi12020137

**Published:** 2021-01-27

**Authors:** Ying Chen

**Affiliations:** 1 Hubei Engineering Technology Research Center of Energy Photoelectric Device and System, Hubei University of Technology, Wuhan 430068, China; chenyddc@163.com; 2 Hubei Collaborative Innovation Center for High-Efficient Utilization of Solar Energy, Wuhan 430068, China; 3 School of Science, Hubei University of Technology, Wuhan 430068, China

**Keywords:** WS_2_ flakes, tungstic acid, chemical vapor deposition, photodetectors

## Abstract

2D WS_2_ is a promising candidate for the next generation nanoelectronics, spintronics, valleytronics, and optoelectronics. However, the uncontrollably large-area growth of WS_2_ nanosheets and their unsatisfactory performance of the photodetectors based on WS_2_ hindered its applications. Here, we proposed a CVD method using tungstic acid as the precursors to grow WS_2_ flakes. After being characterized by AFM, Raman, PL, and TEM, we found the as-grown WS_2_ flakes were high-quality structures. Then the photodetectors based on the as-grown WS_2_ were fabricated, which exhibited high responsivity (7.3 A W^−1^), a fast response rate (a response time of 5 ms and a recovery time of 7 ms), prefect external quantum efficiency (EQE) (1814%), and remarkable detectivity (D*) (3.4 × 10^12^ Jones). Our works provided a new CVD method to grow some high-quality WS_2_ nanosheets.

## 1. Introduction

Atomically thin tungsten disulfide (WS_2_), a 2D crystal with some interesting and important properties, is a promising candidate for the next generation of nanoelectronics, spintronics, valleytronics, and optoelectronics [1,2,3,4,5,6,7]. For example, WS_2_ has a direct bandgap in the visible range and high absorption relative to its thickness [5,8]. WS_2_ exhibits ambipolar field-modulation behavior [9]. The theoretical calculations predict that it has a reduced effective mass, allowing higher carrier mobility [10]. Monolayer WS_2_ has strong PL emission efficiency, stronger than other TMDCs [11,12]. WS_2_ exhibits strong spin-orbit coupling and band splitting due to spin enabling spintronics/valleytronics [13,14,15,16]. WS_2_ also has high nonlinear susceptibility, suggesting its use for nonlinear optical devices [17,18]. Nevertheless, most researchers on WS_2_ are largely limited because of the relatively small lateral size of exfoliated flakes, not to mention the randomness of their shape, thickness, and crystal quality [19,20]. Liquid exfoliation has an advantage for the mass production of WS_2_, but it is difficult to control defects, hindering its application to electronic devices [21,22,23]. CVD is a hopeful approach to grow large-area WS_2_ flakes for extensive device applications. There are two common methods to synthesize WS_2_ by supplying tungsten sources on inert substrates before sulfurization in CVD. One is thin tungsten films or thin tungsten oxide films deposited by various methods including e-beam evaporation, magnetron sputtering, and atomic layer deposition [11,24,25,26,27,28,29]. The other is that of tungsten oxides solid precursors vaporized with solid sulfur sources simultaneously during the CVD process [30,31,32,33,34]. As it is difficult to control the tungsten sources location, distribution, and uniformity precisely, and the poor adhesion of the sources to inert substrates, the controlled CVD growth of large-area uniform monolayer WS_2_ remains a challenge. Some groups used metal substrates (Au) instead of the inert substrates to synthesize a large-area monolayer WS_2_ film; they also tested the electrical properties of WS_2_-based field-effect transistors after bubbling transfer from Au substrates to SiO_2_/Si substrates [35,36,37]. Here we report a new method, spin-coating tungsten acid as a tungsten source on SiO_2_/Si substrates directly before sulfurization, to grow single crystalline WS_2_ monolayers with a size of up to hundreds of micrometers. We also found that photodetectors based on single crystalline WS_2_ flakes have a high responsivity of 7.3 A W^−1^ with a fast response rate of 5 ms, an external quantum efficiency (*EQE*) of 1814%, and a detectivity (D*) of 3.4 × 10^12^ Jones.

## 2. Materials and Methods

The WS_2_ flakes were synthesized by using tungstic acid on SiO_2_/Si substrates as the precursors and sulfurizing via a chemical vapor deposition (CVD) method. First, 2.8 g of tungstic acid (99.9%, Alfa) was dispersed in the oxalic acid solution (0.15 M/L) by ultrasound. Then the tungstic acid colloidal solution was dispersed on a clean SiO_2_/Si substrate by spin-coating. After that, the whole substrate was annealed in air at 100 °C for 1h. Finally, the SiO_2_/Si substrates with tungstic acid were sulfurized by CVD. For CVD growth, the Si/SiO_2_ substrates with the tungstic acid colloidal solution were placed at the center of the quartz tube, and the 0.1 g S powders (99.5%, Alfa) were placed on the upstream side of the Si/SiO_2_ substrates. The quartz tube was flushed with Ar (5N) gas several times and purged to 0.1 Pa with a mechanical pump. The furnace temperature was raised to 900 °C in 30 min and kept for 30 min with a flow rate of 50 sccm Ar gas. After growth, the furnace was naturally cooled to room temperature. The synthesized WS_2_ samples were characterized by an atomic force microscope (AFM, SPM9700, Shimadzu), Raman spectroscopy, and a transmission electron microscope (TEM, Tecnai G^2^ F30 S-TWIN, FEI). The Raman spectra were recorded in the backscattering geometry at a 532 nm line with an argon ion laser Raman spectrometer (LabRAM HR800, Horiba JobinYvon). The Raman mappings were collected by alpha300 R, WITec GmbH, Ulm, Germany, the laser wavelength was 532 nm, and the scanning step interval was 300 nm.

We fabricated the photodetectors based on the WS_2_ flakes by a standard photolithography procedure (MDA-400M, Midas). The 10/50 nm Ti/Au electrode patterns were deposited by an E-beam deposition system (Nexdep, Angstrom Engineering). The photodetector measurement devices contained a broadband laser-driver light source (LDLS, EQ-1500, Energetiq) calibrated by a UV-enhanced silicon photodiode in an ambient atmosphere, a semiconductor characterization system (4200-SCS, Keithley), and an oscilloscope (DSO-X 3052A, Keysight). The oscilloscope light pulse chopped was 500 nm, and the frequency was 3 Hz.

## 3. Results and Discussion

**Figure 1:**
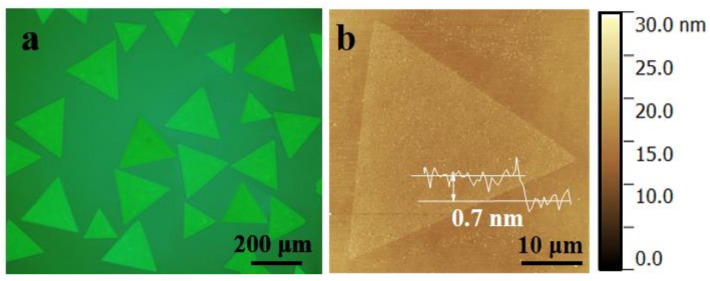
(**a**) Optical microscope images of the monolayer WS_2_. (**b**) AFM images of the monolayer WS_2_.

Figure 1a shows the optical image of the as-grown triangle WS_2_ flakes on SiO_2_/Si substrate; the majority of the flakes were more than 220 μm. The thickness of these triangle crystals was measured by atomic force microscopy (AFM) (Figure 1b). The AFM step height of the WS_2_ flake was typically measured at 0.7 nm, which corresponds to a one-layer structure.

**Figure 2:**
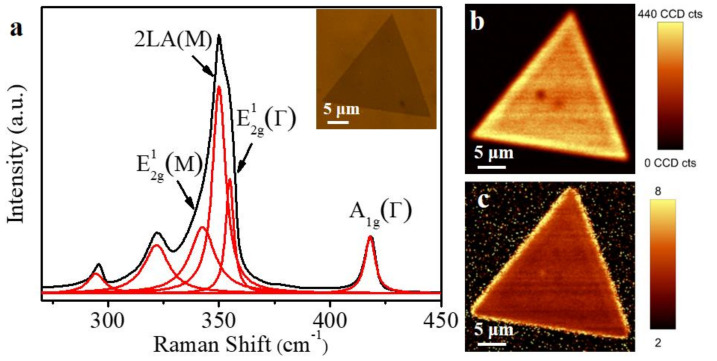
(**a**) Raman characterization of the monolayer WS_2_ triangle flake with 532 nm excitation wavelengths. (**b**) A1g(Γ) intensity mapping for the WS_2_ triangle flake. (**c**) Intensity ratio mapping of 2LA(M) over A1g(Γ) for the WS_2_ triangle flake.

We have further investigated the layer number of the resulting atomically thin WS_2_ triangles by Raman spectroscopy. As shown in Figure 2a, the strongest peak of the WS_2_ triangle flake at about 350 cm^−1^ includes three subpeaks, which are resolved by a multipeak Lorentzian fitting. According to the calculated phonon dispersion [38] and experimental studies [13,39,40,41] of 1L-WS_2_, the in-plane vibrational E^1^_2g_(M) mode peak is at 343 cm^−1^, the second-order mode of longitudinal acoustic phonon 2LA (M) peak is at 350 cm^−1^, and the in-plane vibrational E^1^_2g_(Γ) mode peak is at 355 cm^−1^, respectively. The other peaks at 418, 295, and 322 cm^−1^ are attributed to the out-of-plane A_1g_(Γ) mode, the combination modes of 2LA(M)-2E^2^_2g_(Γ), and the combination modes of 2LA(M)-E^2^_2g_(Γ), respectively. The spectral fingerprint of a monolayer WS_2_ is the frequency separation of 62 cm^−1^ between the E^1^_2g_(Γ) mode and the A_1g_(Γ) mode [11]. Raman images (Figure 2b,c) plotted by extracting the intensity were acquired to demonstrate the uniformity of our WS_2_ samples. The 2LA(M) phonon mode was much more intense than the A_1g_(Γ) mode. The as-grown WS_2_ triangle flake was uniform.

**Figure 3:**
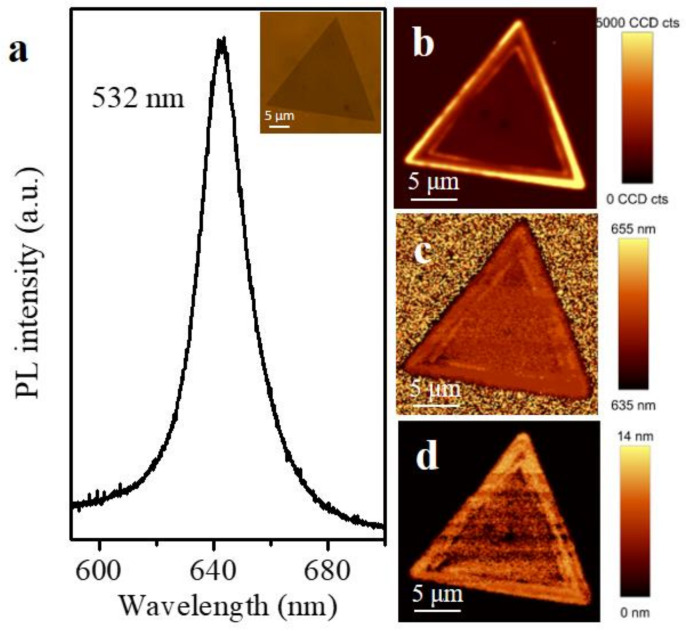
(**a**) PL spectra of the monolayer WS_2_ triangle flake. (**b**–**d**) PL images of the peak integrated intensity, position, and width, respectively.

The optical properties of the monolayer WS_2_ triangle flakes were further investigated by microphotoluminescence (PL). The monolayer WS_2_ flake has a PL peak at about 642 nm, and its full width half maximum (FWHM) value is 22 nm (Figure 3a). The corresponding PL peak-integrated intensity, position, and width mappings of the monolayer WS_2_ triangle flake are shown in Figure 3b–d, respectively. The results of the PL indicated the as-grown WS_2_ was defect free and uniform.

**Figure 4:**
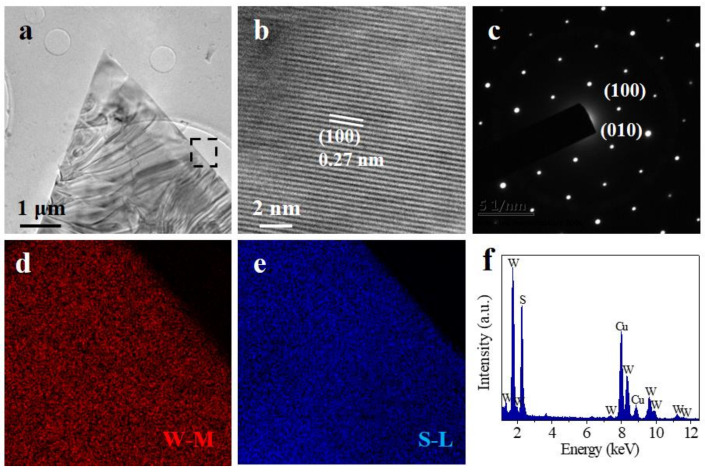
(**a**) Low-magnification TEM image of the WS_2_ triangle flake. (**b**) High-resolution TEM image and (**c**) SAED pattern image of the WS_2_ triangle flake. (**d**,**e**) W and S elemental mapping in the black rectangle region of the WS_2_ flake in (**a**,**f**) EDX spectrum of the WS_2_ flake.

The structures of the monolayer WS_2_ triangle flakes were characterized by TEM. Figure 4a shows the low magnitude bright-field TEM image of a WS_2_ triangular crystal. The high-resolution TEM (HRTEM) image (Figure 4b) shows the hexagonal lattice fringes, which indicates a perfect atomic structure with a lattice spacing of 0.27 nm, corresponding to the (100) planes. The corresponding selected area electron diffraction (SAED) pattern (Figure 4c) revealed the defect free nature of the WS_2_ flake [36]. Then the elemental compositions of the WS_2_ triangle flakes were acquired by EDX mapping and EDX spectrum. EDX mapping in Figure 4d,e indicates the W and S elements distribute evenly. EDX spectrum in Figure 4f demonstrates the atomic ratio of W and S is around 1:2, which is consistent with the original stoichiometry of WS_2_. The TEM results further prove that as-grown WS_2_ flakes are defect free.

**Figure 5:**
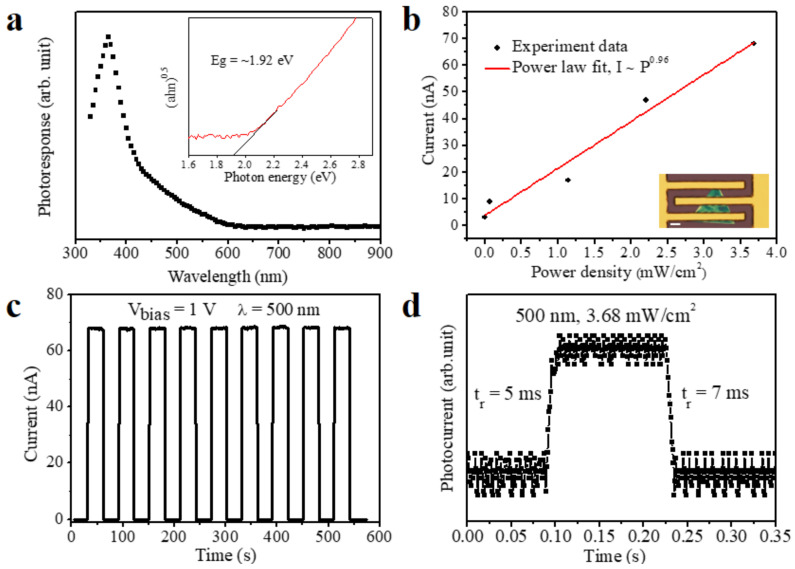
Optoelectronic properties of the WS_2_ triangle flake. (**a**) The spectral response curve of the WS_2_ triangle flake. Inset: the fitting curve of obtaining the bandgap. (**b**) Photocurrent as a function of illumination intensity at V_bias_ = 1 V. Inset: the optical image of the WS_2_ photodetector; the scale bar is 10 μm. (**c**) Time-resolved photoresponse of the WS_2_ photodetector under a bias voltage of 1 V and illumination power of 3.68 mW·cm^−2^. (**d**) Response and recovery curves.

To research the optoelectronic properties of the WS_2_ flakes, photodetectors based on them were fabricated. The spectral response curve reaches a minimum at the wavelength of ≈645 nm in Figure 5a. Hence, the bandgap is about 1.92 eV by calculation, which is consistent with the PL results. Fitting the plot of photocurrent *I*_ph_ on light intensity *P* for the WS_2_ flake as *I*_ph_ ≈ *P*^θ^ obtains the value of θ ≈ 0.96 (Figure 5b), hinting the as-grown WS_2_ has very few defects or traps to photo-induced electron/hole pairs in the test power density range [42]. We further tested the cyclability of the photodetector under 500 nm incident light with the light on/off time interval of 30 s under a bias of 1 V (Figure 5c). The performances of the photodetector are stable. The response and recovery rates were 5 ms and 7 ms tested by an oscilloscope, respectively (Figure 5d). The photoresponsivity was 7.3 A W^−1^, according to the *R*_λ_ = *I*_ph_/*PS*, where *I*_ph_ is the photoexcited current, *P* is the light power intensity, and *S* is the effective area of the photodetector. The external quantum efficiency (EQE) was 1814%, according to the EQE = *hcR*_λ_/*e*λ, where *h* is the Plank’s constant, *c* is the light velocity, *R*_λ_ is the photoresponsivity, *e* is the elementary electronic charge, and λ is the exciting wavelength. The specific detectivity (D*) was 3.4 × 10^12^ Jones, according to the D* = *R*_λ_*S* ^1/2^/(2*eI*_dark_)^1/2^, where *I*_dark_ is the dark current. The performance of our photodetector is more inspiring than the most reported WS_2_ based photodetectors shown in Table 1.

## 4. Conclusions

In summary, we grew the monolayer WS_2_ triangle flakes via a CVD method with the tungstic acid, and researched the performance of the photodetectors based on them. The utilization of tungstic acid colloidal solution could improve the uniformity of the tungsten sources on the substrates. The as-grown monolayer WS_2_ flakes have a size of about 220 μm, a bandgap of about 1.92 eV, and no defects. The photodetectors based on them showed excellent performance, such as high responsivity of 7.3 A W^−1^, large EQE of 1814%, and a fast response rate of 5 ms.

## Figures and Tables

**Table 1:** Comparison of the key parameters of our photodetector.

Photodetectors	Fabrication Method	R_λ_ (A/W)	EQE (%)	Response Time (ms)	Reference
Multilayer WS_2_	CVD	92 × 10^−6^	-	5.3	[4]
Multilayer WS_2_	Exfoliated	5.7	1118	<20	[43]
Monolayer WS_2_	CVD	18.8 × 10^−3^	-	<4.5	[44]
Multilayer WS_2_	PLD	0.51	137	4.1 × 10^−3^	[45]
Monolayer WS_2_	CVD	3.07	763	370	[46]
Multilayer WS_2_	Drop casting	145 × 10^−3^	-	153.78	[47]
Monolayer WS_2_	CVD	7.3	1814	5	This work

## Data Availability

No new data were created or analyzed in this study. Data sharing is not applicable to this article.
